# Genome-wide association study of atopic and autoimmune comorbidities in alopecia areata

**DOI:** 10.3389/fimmu.2026.1810658

**Published:** 2026-04-17

**Authors:** Marisol Herrera-Rivero, Yasmina Gossmann, Swapnil Awasthi, Nicole Cesarato, Stephan Ripke, Bettina Blaumeiser, Ulrike Blume-Peytavi, Gerhard Lutz, Silke Redler, Markus M. Nöthen, Regina C. Betz, F. Buket Basmanav

**Affiliations:** 1 Department of Genetic Epidemiology, Institute of Human Genetics, and Institute of Epidemiology and Social Medicine, University of Münster; Joint Institute for Individualisation in a Changing Environment (JICE), University of Münster and Bielefeld University, Münster, Germany; 2 Institute of Human Genetics, University of Bonn, School of Medicine and University Hospital Bonn, Bonn, Germany; 3 Department of Psychiatry and Psychotherapy, Charité Universitätsmedizin Berlin, Berlin, Germany; 4 Stanley Center for Psychiatric Research, Broad Institute of MIT and Harvard, Cambridge, MA, United States; 5 German Center for Mental Health (DZPG), Partner Site Berlin/Potsdam, Berlin, Germany; 6 Center of Medical Genetics, University and University Hospital of Antwerp, Antwerp, Belgium; 7 Department of Dermatology, Venereology and Allergology, Charité-Universitätsmedizin Berlin, Berlin, Germany; 8 Hair and Nail Medicine, Bonn, Germany; 9 Institute of Human Genetics, Medical Faculty and University Hospital Düsseldorf, Heinrich-Heine-University Düsseldorf, Düsseldorf, Germany

**Keywords:** alopecia areata, autoimmune disease, chronic inflammatory disorders, comorbidities, genetics

## Abstract

**Introduction:**

Alopecia areata (AA) is a chronic autoimmune-mediated disorder characterized by hair loss from the scalp and/or body. AA patients frequently present with comorbid chronic inflammatory disorders (CIDs), particularly atopic and autoimmune diseases. Genome-wide association (GWA) studies have suggested a genetic link. However, no studies to date have examined genetic factors that are associated with the comorbid development of CIDs directly in individuals affected by AA.

**Methods:**

We performed an exploratory GWA study in Central European AA patients stratified by self-reported comorbidity status (110 to 1,302 cases with- and 1,030 controls without comorbid CIDs). Comorbidities were analyzed first as broad atopic and autoimmune categories and subsequently as individual conditions, including asthma, atopic dermatitis, rhinitis, vitiligo, and Hashimoto’s thyroiditis.

**Results:**

No genome-wide significant signals were identified at either the variant or gene level. At exploratory thresholds (p_variant_<1x10^-5^, p_gene_<0.001), more loci showed potential association with comorbid autoimmunity than with atopy, although the number of implicated genes was comparable. Several identified genes were previously implicated in CID pathogenesis and many loci contained variants with known regulatory effects on gene expression in skin and immune cells. For comorbid atopy/autoimmunity overall, *PHF11* was the most frequently implicated gene, consistent with its previously described role in T- and B-cell biology. Network analyses highlighted cytokine, hormone, and transcription factor signaling pathways as potential mechanisms underlying comorbid CID development in AA.

**Discussion:**

Our study provides initial mechanistic insights for comorbid CID development in AA, and a foundation for larger-scale studies.

## Introduction

Alopecia areata (AA) is a chronic autoimmune-mediated disease directed against the hair follicle, resulting in sudden, non-scarring hair loss. AA affects all age groups and is one of the most common causes of hair loss in the population, with a lifetime risk of around 2% (1). AA typically begins with the appearance of hairless patches (patchy AA), which may in some patients merge and progress to complete baldness of the scalp (alopecia totalis, AT) and/or body (alopecia universalis, AU) (2). The disease course is highly variable and often chronic, marked by unpredictable relapses and remissions or persistent hair loss without regrowth (2). AA imposes a major psychosocial burden on affected individuals, who are at an increased risk of mental health problems such as major depression and anxiety (3, 4).

AA is associated with an array of further medical comorbidities (5, 6). In particular, epidemiological evidence shows that patients with AA have a higher prevalence and incidence of chronic inflammatory diseases (CIDs), including atopic and autoimmune disorders (7). Moreover, as we have recently shown, comorbidity with particular atopic or autoimmune disorders, such as atopic dermatitis, asthma, rhinitis, Hashimoto’s thyroiditis (HT) and vitiligo, are associated with variability in clinical features of AA, including age-of-onset, severity or duration of the hair loss (8).

AA is regarded as a multifactorial disorder resulting from the interaction between genetic predisposition and environmental triggers, such as psychological stress. To date, genome-wide association (GWA) studies of AA by our group and others have identified associations with ten genetic loci, and found suggestive evidence for an additional four (9, 10). The identified loci implicated genes that participate in innate and adaptive immune responses, as well as in autophagy, apoptosis, pigmentation and oxidative stress. Notably, a number of these AA risk loci appear to overlap with genomic regions previously linked to the risk of atopic diseases and other autoimmune disorders (e.g. *IL4*/*IL13*, *IL15RA*/*IL2RA*, *CTLA4*/*ICOS*), suggesting shared or pleiotropic genetic effects and potentially overlapping molecular mechanisms that may contribute to the co-occurrence of AA with other CIDs.

Genetic relationships between complex traits are commonly evaluated using genetic correlation analyses that estimate shared heritability based on summary statistics from independent GWA studies. This approach can help explain epidemiologically observed comorbid disease patterns by identifying the extent to which conditions share a common genetic basis. However, the interpretation of genetic correlation estimates is complicated by the inherent heterogeneity of the GWA study datasets on which they rely, including differences in study design, phenotype definitions, and ancestry composition. Such sources of variation can introduce bias, reduce statistical power, and obscure disease-specific signals. More importantly, genetic correlation-based approaches do not identify novel genetic variants that may contribute specifically to comorbidity development within a particular disease context, nor can they resolve etiologic heterogeneity between clinically-defined subgroups of patients. Addressing these subgroup-specific questions requires genetic analyses conducted directly within a single, clinically-stratified cohort, an approach that has not yet been undertaken for AA.

To fill this gap and to provide first genetic insights into the comorbid development of CIDs in the context of AA, we conducted exploratory GWA analyses in 2,332 individuals of Central European descent affected by AA. The AA patients were stratified as cases or controls according to their self-reported comorbidity status for CIDs, which were subdivided into two main categories: atopic and autoimmune. Case phenotypes were then defined in a stepwise manner, from general to specific. First, presence of any atopic or autoimmune comorbidity qualified individuals for a “case” classification. Second, atopic diseases and autoimmune disorders were analyzed as separate general categories. Finally, individual diseases with at least 100 affected patients were examined, including atopic dermatitis, rhinitis, asthma, vitiligo, autoimmune thyroid disease (AITD) in general, which comprises HT and Graves’ disease, and HT specifically. Analyses were conducted at both the variant and gene levels using liberal thresholds established for exploratory purposes, and the identified loci were subsequently subjected to gene enrichment and network analyses to biologically contextualize the findings.

## Methods

### Study sample

The present study was performed using data from a cohort of individuals with AA (N = 2,631) and Central European ancestry who were recruited at outpatient clinics, dermatology practices, and AA self-support groups in Germany and Belgium between 2000 and 2023 (8).

Demographic and clinical data were collected from all participants using a self-report questionnaire, completed either by the patients themselves or, in the case of minors, by their parents (8). The questionnaire contained items on the clinical course and family history of AA, presence of a list of specified CIDs as well as any additional comorbidities not listed, and responses to a set of predefined AA treatments. The specified CIDs comprised asthma, atopic dermatitis, rhinitis, type I diabetes mellitus, vitiligo, thyroid disease and the specific type thereof, Crohn’s disease, and ulcerative colitis.

The comorbid presence of atopic and autoimmune diseases was determined based on pooled information from both the section of the questionnaire addressing the listed CIDs and the section asking about any additional, non-specified comorbid conditions.

A total of 2,469 individuals with phenotypic and genetic data were initially considered for inclusion in the present study. Of these, 39 were excluded due to missing information on CID comorbidity status.

The study was performed in accordance with the principles of the Declaration of Helsinki. Ethical approval was obtained from the ethics committee of the Medical Faculty of the University of Bonn. Written informed consent was obtained from all patients or legal guardians prior to inclusion.

### Genotype data

Genotype data for the AA cohort described here were generated in three independent genotyping batches. Genotyping and quality control (QC) procedures for two of these datasets have been published elsewhere (referred to as Central Europe 1 and Central Europe 2 case cohorts in 10; n= 1,280). For the remainder of the cohort (n= 1,189), genotyping and QC were performed within the context of the present study following similar procedures.

Briefly, genome-wide genotyping of all AA cases investigated in the present study was performed in three batches using either the Human660W-Quad v1.0 (2009-2010), HumanOnmiExpress-12 v1.0 (2010), and Infinium Global Screening Array-24 v2.0 (2019), or v3.0 (2022) arrays (Illumina). Each batch included samples from population-based cohorts as healthy controls, as described previously (10). In the present study, only genotype data from individuals affected by AA were analyzed. Population-based control samples included in each genotyping batch as part of the original study design (i.e. case-control framework) (10) were used only for the initial two rounds of quality control (QC) as detailed below, and were not considered in subsequent analyses.

In detail, the first round of QC involved in each batch separately the exclusion of: (i) variants with a missingness rate >5% before and >2% after sample removal; (ii) variants with difference in missingness rate >2% between cases and controls; (iii) variants with a Hardy-Weinberg equilibrium (HWE) p<1x10^–6^ in controls or p<1x10^–10^ in cases and (iv) samples with a missingness rate >2%; (v) and/or autosomal heterozygosity deviation (|Fhet|>0.2). Genotype imputation was also carried out separately in each batch using a stepwise pre-phasing and imputation approach, as implemented in Eagle2 (11) and Minimac3 (12) using default parameters and a variable chunk size of 132 genomic chunks. The imputation reference included 54,330 phased haplotypes, with 36,678,860 variants from the Haplotype Reference Consortium (HRC) panel. Relatedness testing and population structure analyses were conducted using a subset of SNPs that passed stringent post-imputation quality control (i.e. INFO > 0.8, missingness < 1%, and minor allele frequency > 0.05). These variants were further LD-pruned (r² < 0.02) during the second round of QC. For cryptically related individuals (i.e. pi-hat > 0.2), one individual from each related pair was removed at random, with preferential retention of cases over controls. Best-guess genotype was obtained by calling the genotype with posterior probability greater than 0.8, with calls set to missing when no genotype exceeded this threshold. Furthermore, after excluding variants with an imputation INFO metric of <0.8 and retaining only the AA cases (i.e. excluding data from population based controls), individuals from all three datasets were merged. A new round of QC was performed for the merged dataset using the plinkQC R package to ensure robustness. Here, the following were excluded: (i) variants with missingness rate >10%, minor allele frequency <1%, and HWE p<1x10^–5^ and (ii) individuals with no phenotypic data or failed heterozygosity.

The final dataset comprised the genotypes of 2,332 AA affected individuals and 5,180,826variants. The genotyping rate was 0.996. Principal components analysis (PCA) of the combined cohort showed no clustering of AA individuals by the three independent datasets (e.g., no genotyping batch effect), and low variability across the study population (5%). Potential stratification due to ancestry and/or technical artifacts was adequately captured within the first 20 principal components (**Supplementary Figure 1**).

### Cohort stratification and phenotype definitions

Individuals in the AA cohort were classified into case and control groups according to the self-reported comorbid presence of CIDs. Case classifications followed a hierarchical scheme previously applied in our earlier study (8). This approach organized comorbidities from broad categories of related diseases to more specific individual conditions that were reported in at least 100 individuals.

In the present study, self-reported comorbid CIDs were initially classified under two general categories as atopic or autoimmune. The atopic category comprised three common atopic disorders: atopic dermatitis, rhinitis and asthma. All remaining CIDs reported in the cohort were classified as autoimmune, encompassing both common and rare immune-mediated diseases with varying levels of evidence supporting an autoimmune etiology (e.g., vitiligo and psoriasis, representing high and intermediate levels of evidence, respectively). The majority of autoimmune comorbidities were represented by a limited number of conditions, including autoimmune thyroid disease, vitiligo, psoriasis, rheumatoid arthritis, inflammatory bowel disease, and type 1 diabetes. Among AA patients reporting at least one autoimmune comorbidity, over 90% had at least one of these conditions. The remaining autoimmune diseases were rare and each occurred only in small numbers of individuals.

The control group comprised AA patients without comorbid CIDs (n=1,030). To capture multiple levels of comorbidity specificity, ten primary case phenotypes were defined for the GWA analyses, as shown in **Table 1**. These included four broad case phenotypes defined in accordance with the comorbid presence of:

**Table 1:** Demographic and clinical characteristics and comorbidity-stratified phenotype definitions of the AA study cohort.

Characteristic	Value	Sample
N	2,332	Total
Females (%)	1,751 (75.0)	Total
Age-at-onset (mean ± SD)	27.91 ± 17.2	Total
Age-at-recruitment (mean ± SD)	40.89 ± 15.78	Total
*Alopecia areata type*		
Patchy (%)	1,223 (52.4)	Total
Alopecia Totalis - AT (%)	186 (8.0)	Total
Alopecia Universalis - AU (%)	169 (7.2)	Total
AT/AU (%)	793 (34.0)	Total
Comorbidity-stratified phenotype definitions		
No comorbidities (%)	1,030 (44.2)	Control cohort
Any atopic/autoimmune comorbidity (%)	1,302 (55.8)	Case cohort 1
Atopic diseases only (%)	889 (38.1)	Case cohort 2
Autoimmune diseases (AIDs) only (%)	220 (9.4)	Case cohort 3
Atopic + AID (%)	192 (8.2)	Case cohort 4
Atopic dermatitis (%)	646 (27.7)	Case cohort 5
Atopic dermatitis without AIDs (%)	537 (23.0)	*Follow-up 1 – Case 5*
Atopic dermatitis without other diseases (%)	254 (10.9)	*Follow-up 2 – Case 5*
Rhinitis (%)	653 (28.0)	Case cohort 6
Rhinitis without AIDs (%)	538 (23.1)	*Follow-up 1 – Case 6*
Rhinitis without other diseases (%)	231 (9.9)	*Follow-up 2 – Case 6*
Asthma (%)	336 (14.4)	Case cohort 7
Asthma without AIDs (%)	270 (11.6)	*Follow-up 1 – Case 7*
Asthma without other diseases (%)	50 (2.1)	*Follow-up 2 – Case 7*
Vitiligo (%)	110 (4.7)	Case cohort 8
Autoimmune thyroid disease - AITD (%)	175 (7.5)	Case cohort 9
AITD without atopic diseases (%)	94 (4.0)	*Follow-up 1 – Case 9*
Hashimoto’s thyroiditis – HT (%)	153 (6.6)	Case cohort 10
HT without atopic diseases (%)	79 (3.4)	*Follow-up 1 – Case 10*

- ≥ 1 CID of any type (any atopic/autoimmune comorbidity; case cohort 1);
- ≥ 1 CID of a single type only, that is atopic only (case cohort 2) or autoimmune only (case cohort 3); and
- ≥ 1 CID of both types simultaneously (atopic + autoimmune; case cohort 4).

In addition, six disease-specific case phenotypes (case groups 5–10) were defined for individual comorbid disorders reported by at least 100 AA patients. These comprised atopic dermatitis, rhinitis, asthma, vitiligo, AITD overall (encompassing HT and Graves’s disease) and HT. For each disease-specific phenotype, all individuals reporting the respective comorbid disease were classified as cases for that phenotype, regardless of whether they also reported additional atopic or autoimmune comorbidities. Patients reporting comorbid conditions present in fewer than 100 individuals in the AA cohort (e.g., type 1 diabetes, rheumatoid arthritis) were included as cases in the broader phenotype categories 1, 3, and/or 4.

### More stringent case definitions for individual diseases: follow-up analyses

We reasoned that the presence of additional comorbidities in some individuals within the disease-specific groups could confound the detection of disease-specific genetic association signals in the respective GWA analyses. To address this possibility, variants identified in the disease-specific GWA analyses (GWA 5-10) were re-tested for their association with the respective disease by follow-up analyses using stricter case definitions designed to better isolate each disease. Hereby, two follow-up models were applied. In model 1, which represents the less stringent approach, cases were defined as AA patients with the respective atopic or autoimmune disease who did not report any comorbidities from the other class. In model 2, representing the more stringent approach, cases were required to have the respective disease only and to report no additional comorbidities of either category. Follow-up analyses were performed only when at least 50 individuals met the corresponding case definition. Consequently, model 1 follow-up analyses were conducted for atopic dermatitis, rhinitis, asthma, AITD and HT, while model 2 follow-up analyses were feasible for atopic dermatitis, rhinitis and asthma (**Table 1**).

### Variant-based GWA analyses

In total, ten primary GWA analyses were conducted in the current study, each comparing AA patients with a particular comorbid profile to AA patients without comorbidities. All variant-based analyses were performed using Plink 2.0 (13). Here, additive logistic regression models were applied, adjusting for the following covariates: sex, age-at-AA onset, age-at-recruitment, AA type, family history of AA, and the first 20 principal components from a genomic PCA. Given the exploratory character of our study, we adopted the commonly accepted thresholds of statistical significance for genome-wide (p<5x10^-8^) and suggestive (p<1x10^-5^) findings in GWA studies. For the follow-up analyses, a nominal confirmatory significance threshold of p<0.01 was applied in view of the limited number of variants tested.

### Functional mapping and annotation analyses

To provide a genomic context for the variant-based findings, the summary statistics were subjected to locus analysis and variant annotation using the SNP2GENE tool of the FUMA GWAS platform (14). Here, genomic loci with suggestive associations (p<1x10^-5^) to the investigated phenotypes were identified. Linkage disequilibrium (LD) clumping was performed on input variants with p<0.05 using an r≥0.6 in a window of 500 kb, in accordance with the European population reference panel of the 1000 Genomes Project. Variants in the reference panel that were not present in our dataset were excluded.^2^ This approach identified genomic loci led by suggestive-threshold genetic variants that included nominal-threshold variants in moderate LD. Positional gene and expression quantitative trait loci (eQTL) mappings were then performed according to a maximum distance of 1 kb from the respective gene boundaries in hg19 coordinates and for the following gene types: protein-coding, long non-coding RNA (lncRNA), non-coding RNA (ncRNA), and processed transcripts. For eQTL mapping, we queried all tissues from the following datasets available within the FUMA GWAS platform: van der Wijst et al. scRNA eQTLs (immune cells), DICE (immune cells), Blood eQTLs, GTEx v8 Adipose Tissue, GTEx v8 Blood, GTEx v8 Blood Vessel, and GTEx v8 Skin. Details are available from the FUMA GWAS website (https://fuma.ctglab.nl/). Only variant-gene pairs with a false discovery rate (FDR) <0.05 were annotated.

### Gene-based GWA analyses

In addition to the identification of genomic loci via the suggestive association of single variants and LD clumping, genomic loci were also identified using gene-based GWA analyses. Variant-level summary statistics were subjected to gene analysis using the Multi-marker Analysis of GenoMic Annotation (MAGMA) software (15). Gene boundaries were defined as the start and end positions ±1 kb, according to the Ensembl’s hg19 genome build. In total, 18,497 genes were present in our dataset for this analysis. For exploratory purposes, the genome-wide and suggestive significance thresholds were set to p<2.7x10^-6^ (0.05/18497) and p<0.001, respectively.

### Gene set enrichment and protein-protein interaction network analyses

To inform the potential biological meaning of the genetic findings, we performed protein-protein interaction (PPI) network analyses by creating three PPI networks from the following inputs: a) atopic/autoimmune comorbidities: all genes mapped by location and known eQTLs to all identified genomic loci, together with all genes resulting from the MAGMA analyses, for all phenotypes studied; b) atopic comorbidities: genes mapped by location and known eQTLs to identified genomic loci, together with genes resulting from the MAGMA analyses, for asthma, atopic dermatitis, rhinitis, and atopic (only) phenotypes; and c) autoimmune comorbidities: genes mapped by location and known eQTLs to identified genomic loci, together with genes resulting from the MAGMA analyses, for vitiligo, AITD, HT, and autoimmune (only) phenotypes. These networks were generated by incorporating linker proteins (i.e., non-input proteins that create indirect links between input proteins) and functional interaction annotations using the Gene Set analysis tool of the ReactomeFIViz app for Cytoscape v.3.9.1 (16, 17). Subsequently, an enrichment analysis of pathways for each network was performed, which included input and linker proteins. Initially, gene sets with FDR<0.05 were considered significantly enriched. These were then filtered to retain the top 100 terms for each network using the following criteria: a) FDR<0.01, b) overlap between at least three network proteins and the pathway proteins, and c) the term is not associated with a specific disease. Excluded disease-associated terms included hepatitis B, shigellosis, prostate cancer, type II diabetes mellitus, AGE-RAGE signaling pathway in diabetic complications, and proteoglycans in cancer.

## Results

### Study cohort and case-control phenotypes

The final study sample comprised 2,332 individuals with AA. Of these 2,332 individuals, 1,751 (75%) were female. Mean age-at-AA onset was 28 (± 17) years and the most frequent disease type was patchy AA (52%). Mean age-at-recruitment was 41 (± 16) years.

In total, 1,030 (44.2%) individuals reported none of the studied atopic/autoimmune comorbidities and were classified as controls, and 1,302 (55.8%) reported at least one of the investigated comorbidities and were allocated to the various case groups.

A total of 889 (38.1%) individuals reported ≥ 1 of the three investigated common atopic diseases (i.e., atopic dermatitis, rhinitis, and asthma) with no other autoimmune disorders; 220 (9.4%) reported comorbid autoimmune diseases only; and 192 (8.2%) reported both atopic and autoimmune comorbid disorders. Rhinitis and atopic dermatitis were the most frequently reported comorbidities (approximately 28% of the sample, respectively). The effective group sizes for all of the investigated phenotypes are shown in **Table 1**.

### Genetic loci identified by variant-based GWA analyses

**Figure 1:**
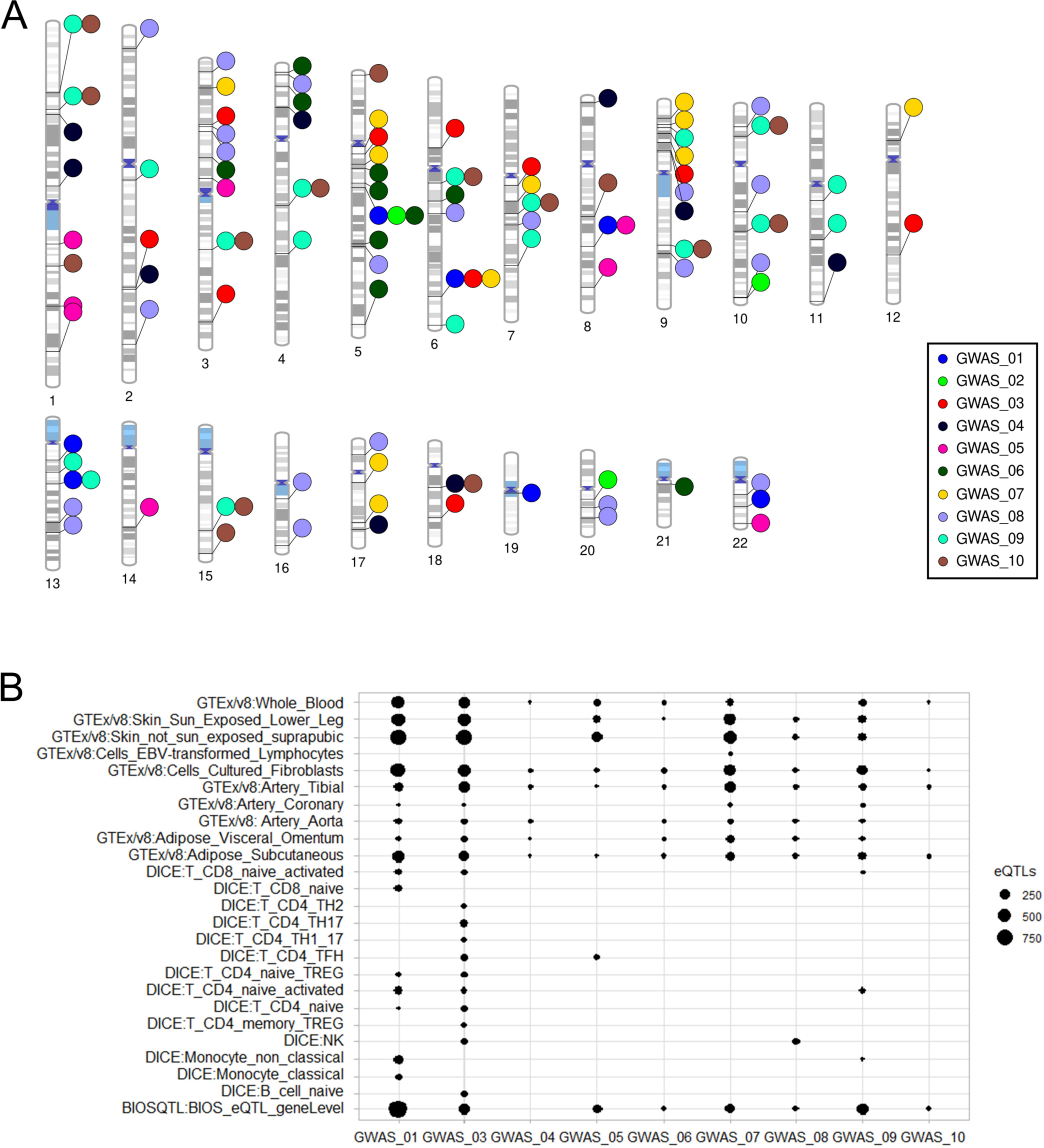
Summary of results obtained from functional mapping and annotation analyses using SNP2GENE. **(A)** Phenogram of suggestive (p<1x10^-5^) genomic loci. Details are provided in **Supplementary Table 1**. **(B)** Number of annotated expression quantitative trait loci (eQTLs) per tissue. There were no annotations for the atopic (only) phenotype (GWAS_02). The phenotypes corresponding to GWAS IDs are provided in **Table 2**. GWAS_01= atopic/autoimmune (any); GWAS_02= atopic (only); GWAS_03= autoimmune (only); GWAS_04= atopic + autoimmune; GWAS_05= atopic dermatitis; GWAS_06= rhinitis; GWAS_07= asthma; GWAS_08= vitiligo; GWAS_09= AITD; GWAS_10= HT. AITD: autoimmune thyroid disease, HT: Hashimoto’s thyroiditis.

Ten variant-based GWA analyses were conducted, each comparing the non-comorbid AA control group with a distinct comorbid AA patient subgroup, defined according to the presence of comorbid CIDs (e.g., atopic/autoimmune [any], atopic [only], autoimmune [only], or specific comorbidities). None yielded genome-wide significant associations (p<5x10^-8^). At the suggestive (p<1x10^-5^) threshold, between three (atopic [only]) and 22 (vitiligo) genomic loci showed a potential association with atopic and/or autoimmune comorbidities in individuals with AA (**Table 2**; **Supplementary Table 1**). Notably, some of the loci identified in these analyses comprised only a single variant. Therefore, we considered that loci with >1 variant were the most likely to represent true association signals. Of 114 loci, 99 were supported by at least one other variant. In general, more suggestive loci were found for autoimmune than for atopic phenotypes, even after accounting for signal support. Autoimmune phenotypes also showed stronger association signals than atopic phenotypes in terms of lower p-values and larger effects, as defined by the odds ratio (OR). For the atopic/autoimmune comorbidities (any) phenotype, the strongest association signal was found in chr13:50009874-50108857 (locus #5, lead variant: rs3794381, p=2.5x10^-6^, OR = 1.4, mapped gene: *PHF11*; **Supplementary Figure 2**). A summary of the results from the locus analysis is provided in **Table 2** and **Figure 1A**. Details of all identified loci are provided in **Supplementary Table 1**.

**Table 2:** Summary of variant and gene-based GWA results obtained for all phenotypes.

Item	Atopic/autoimmune (any)	Atopic (only)	Autoimmune (only)	Atopic + autoimmune	Atopic dermatitis	Rhinitis	Asthma	Vitiligo	AITD	HT
Analysis ID for figures	GWAS_01	GWAS_02	GWAS_03	GWAS_04	GWAS_05	GWAS_06	GWAS_07	GWAS_08	GWAS_09	GWAS_10
# Loci (suggestive, p<1x10^-5^)	7	3	10	9	8	10	11	22	19	15
# Supported loci (variants>1)	7	3	8	7	8	9	10	21	14	12
# Variants in loci (p<0.05, LD)	329	13	400	113	216	159	261	325	255	127
# Mapped genes	12	0	26	7	9	7	11	41	27	21
Top locus	13:50009874-50108857	10:132690980-132697051	6:149643709-149786639	8:3051115-3065803	8:102491136-102494523	3:67087852-67150590	6:149643709-149786639	20:42702424-42790823	10:20731957-20754266	3:130647261-131335525
Best variant	rs3794381	rs4704604	rs6926771	rs17079577	rs6999959	rs62259081	rs62426122	rs6103623	rs78382211	rs74421728
Best p-value	2.53E-06	3.96E-06	4.24E-07	3.95E-07	9.03E-07	1.14E-06	3.09E-06	1.39E-07	2.14E-07	4.21E-07
Best odds ratio	1.4	1.406	1.92	1.99	0.49	1.92	1.62	4.2	6.71	6.45
Best mapped/nearest gene	*PHF11*	*MIR378C*	*TAB2*	*CSMD1*	*KB-1562D12.1*	*KBTBD8*	*TAB2*	*JPH2*	*AMD1P1*	*ATP2C1*
# Annotated eQTL pairs (FDR<0.05)	3605	0	2985	35	499	122	1791	242	788	45
# Annotated eQTL genes	21	0	20	3	12	7	16	19	16	5
# Gene-level associations (p<0.001)	16	20	19	13	16	12	21	16	22	21
Top gene (MAGMA)	*PHF11*	*ADH4*	*SYCP3*	*NRCAM*	*LCE5A*	*JAM3*	*SUMO4*	*KBTBD3*	*DDRGK1*	*GTSF1L*
Top gene (MAGMA) p-value	2.39E-05	9.27E-05	2.57E-05	3.41E-05	3.14E-05	1.07E-04	5.89E-06	1.55E-04	1.25E-05	2.81E-05
# Follow-up 1 variants (p<0.01)	–	–	–	–	188	157	253	–	139	62
# Follow-up 2 variants (p<0.01)	–	–	–	–	141	117	8	–	–	

AITD, autoimmune thyroid disease; HT, Hashimoto’s thyroiditis; LD, linkage disequilibrium; eQTL, expression quantitative trait loci; FDR, false discovery rate.

Definitions – LD, r2≥0.6 + distance<500kb; MAGMA, gene-level association analysis; follow-up 1, respective (atopic or autoimmune) disease and no diseases of the other class; follow-up 2, the respective (atopic or autoimmune) disease only and no other diseases.

Some of the identified loci showed suggestive association with more than one phenotype (**Figure 1A**). These comprised: 10 loci for AITD and HT (chr1:47664233-47876421, chr1:59258631-59258631, chr3:130647261-131335525, chr4:96555809-96555809, chr6:71088713-71088713, chr7:86384792-86546681, chr9:99031628-99060185, chr10:20731957-20754266, chr10:86605342-87174443, chr15:80352529-80567419); one locus for atopic/autoimmune comorbidities (any) and atopic dermatitis (chr8:102491136-102494523); one locus for atopic/autoimmune comorbidities (any) and AITD (chr13:50009874-50090844/50108857, partial overlap); one locus for atopic + autoimmune and HT (chr18:34091305-34149939); one locus for atopic/autoimmune comorbidities (any), atopic (only), and rhinitis (chr5:79181467-79197782/79198171, partial overlap); and one locus for atopic/autoimmune comorbidities (any), autoimmune (only), and asthma (chr6:149643709-149786639).

### Positional gene and eQTL mapping of variant-based GWA results

Gene and eQTL annotations returned 194 unique genes that were mapped by genomic location and/or were known to be regulated in the queried tissues (i.e., blood, skin, fibroblasts, and immune cells, as well as vascular and adipose tissues) by variants in identified loci (**Supplementary Table 2**). Of the 10 phenotype categories, only the atopic (only) phenotype showed no gene annotations. The majority of phenotypes showed eQTL annotations for fibroblasts, as well as blood, skin, and arterial and adipose tissues. Only those loci identified for the pooled autoimmune phenotypes (i.e. autoimmune [only]) showed widespread eQTL effects in immune cells. The respective immune cells mainly concerned T cells of various types (i.e., CD8+ T cells, CD4+ T helper [Th 2/17/1_17], and [memory] T regulatory cells [Tregs], and T follicular helper cells [TfH]). However, natural killer (NK) and naive B cells were also implicated. Loci for atopic/autoimmune comorbidities (any) were also annotated with eQTLs in some immune cells, particularly in monocytes, but also in CD4+ and CD8+ T cells (**Figure 1B**). In terms of the individual atopic and autoimmune diseases, immune cell eQTL annotations were identified in TfH cells for atopic dermatitis, NK cells for vitiligo, and CD4 activated T cells and non-classical monocytes for AITD. One of the most interesting genes identified through these annotations was *TAB2*, which corresponded to the locus for atopic/autoimmune comorbidities (any), autoimmune (only) and asthma (chr6:149643709-149786639). Although the lead variant in the region is intergenic, this and a large number of nominally significant variants in strong LD are known regulators of *TAB2* expression in skin (**Supplementary Figure 3**). Accordingly, this gene showed large numbers of mapped variants (65-86) in the locus, as well as eQTLs (95-122) in skin tissues. A summary of the positional gene and eQTL annotation results are provided in **Supplementary Table 2**; **Figure 1B**.

### Follow-up analyses for the single diseases

To address potential confounding by additional comorbid conditions some patients had, variants identified in the disease-specific GWA analyses (GWA 5–10) were re-tested in follow-up analyses using stricter case definitions to better isolate each disease. The less stringent follow-up 1 analyses showed that a large proportion of variants in the loci identified for HT (49%), AITD (54%), atopic dermatitis (87%), rhinitis (99%), and asthma (97%) were nominally associated (p<0.01 as the confirmatory significance threshold) with the respective disorder when no comorbid disease from the other class was present. The more stringent follow-up 2 analyses showed that 65%, 74%, and 3% of the variants in the loci identified for atopic dermatitis, rhinitis and asthma, respectively, were associated at the confirmatory significance threshold with the disease when no other comorbid diseases were present. A summary of the results for both follow-up analyses is provided in **Table 2** (overall) and **Supplementary Table 1** (by locus).

### Gene-based GWA analyses

**Figure 2:**
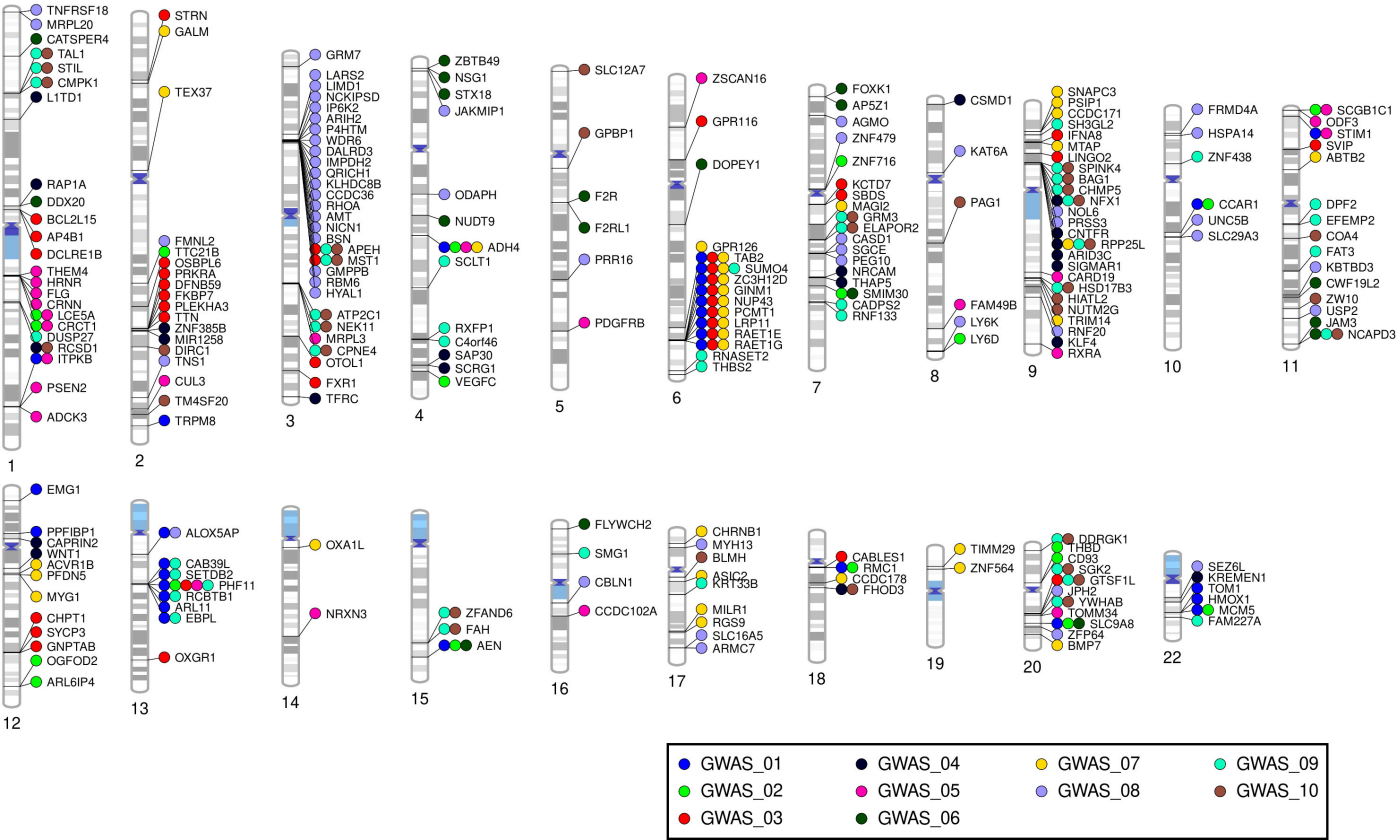
Phenogram presenting a gene-level summary of the present findings. The phenogram shows protein-coding genes mapped by location to suggestive loci (p<1x10^-5^), eQTL genes annotated to suggestive loci, and MAGMA gene associations (p<0.001) obtained for all investigated phenotypes. GWAS_01= atopic/autoimmune (any); GWAS_02= atopic (only); GWAS_03= autoimmune (only); GWAS_04= atopic + autoimmune; GWAS_05= atopic dermatitis; GWAS_06= rhinitis; GWAS_07= asthma; GWAS_08= vitiligo; GWAS_09= AITD; GWAS_10= HT. AITD: autoimmune thyroid disease, HT: Hashimoto’s thyroiditis.

The gene-based analyses generated no genome-wide (p<2.7x10^-6^) association signals. At the adopted exploratory threshold (p<0.001), between 12 (rhinitis) and 22 (AITD) genes showed suggestive association with atopic and/or autoimmune comorbidities in individuals with AA (**Table 2**; **Supplementary Table 3**). The top gene for atopic/autoimmune comorbidities (any) was *PHF11* (Z = 4.1, p=2.4x10^-5^). Moreover, this gene was the most frequent finding across phenotypes, being observed for atopic/autoimmune comorbidities (any), atopic (only), autoimmune (only), atopic dermatitis, and AITD phenotypes (**Figure 2**). In total, 307 genes showed suggestive association with at least one of the studied phenotypes at the adopted significance threshold. Of these, 62 showed cross-phenotype overlap, mainly concerning more general phenotypes paired with specific disorders (e.g., *CRCT1* for atopic [only] and atopic dermatitis; *SETDB2* for atopic/autoimmune comorbidities [any] and AITD; *AEN* for atopic/autoimmune comorbidities [any], atopic [only], and rhinitis). As expected, extensive gene overlaps were found between AITD and HT (30 genes, e.g. *TAL1*, *STIL*, *NEK11*, *MST1*, *ELAPOR2*, *BAG1*) but, in general, only limited gene overlaps were found between single diseases. Besides *PHF11*, genes shared across individual disease phenotypes comprised *ADH4* (asthma and atopic dermatitis), *RPP25L* (asthma, AITD, and HT), *NCAPD3* (rhinitis, AITD, and HT), and *SUMO4* (asthma and AITD). Results of the MAGMA gene-based analyses are summarized in **Table 2** and **Figure 2**. Detailed results are provided in **Supplementary Table 3**.

### Gene-set enrichment and protein-protein interaction network analysis

Finally, gene-level results from the variant- and gene-based analyses were integrated in order to generate an improved overview of the implicated biological processes. First, we used the GENE2FUNC tool from FUMA GWAS to perform gene set enrichment analyses, but this returned no significant functional terms for our gene lists. In two non-functional categories, the following enrichments (FDR<0.05) were found: i) eight positional gene sets (chr3p21, chr9p13, chr2q31, chr6q25, chr9p22, chr6q27, chr13q14 and chr1p33); and ii) GWAS Catalog traits including Ulcerative colitis, Crohn’s disease, Inflammatory bowel disease, Coronary artery aneurysm in Kawasaki disease, Sleep duration (short sleep), and Depressive symptoms (binary sum-score).

**Figure 3:**
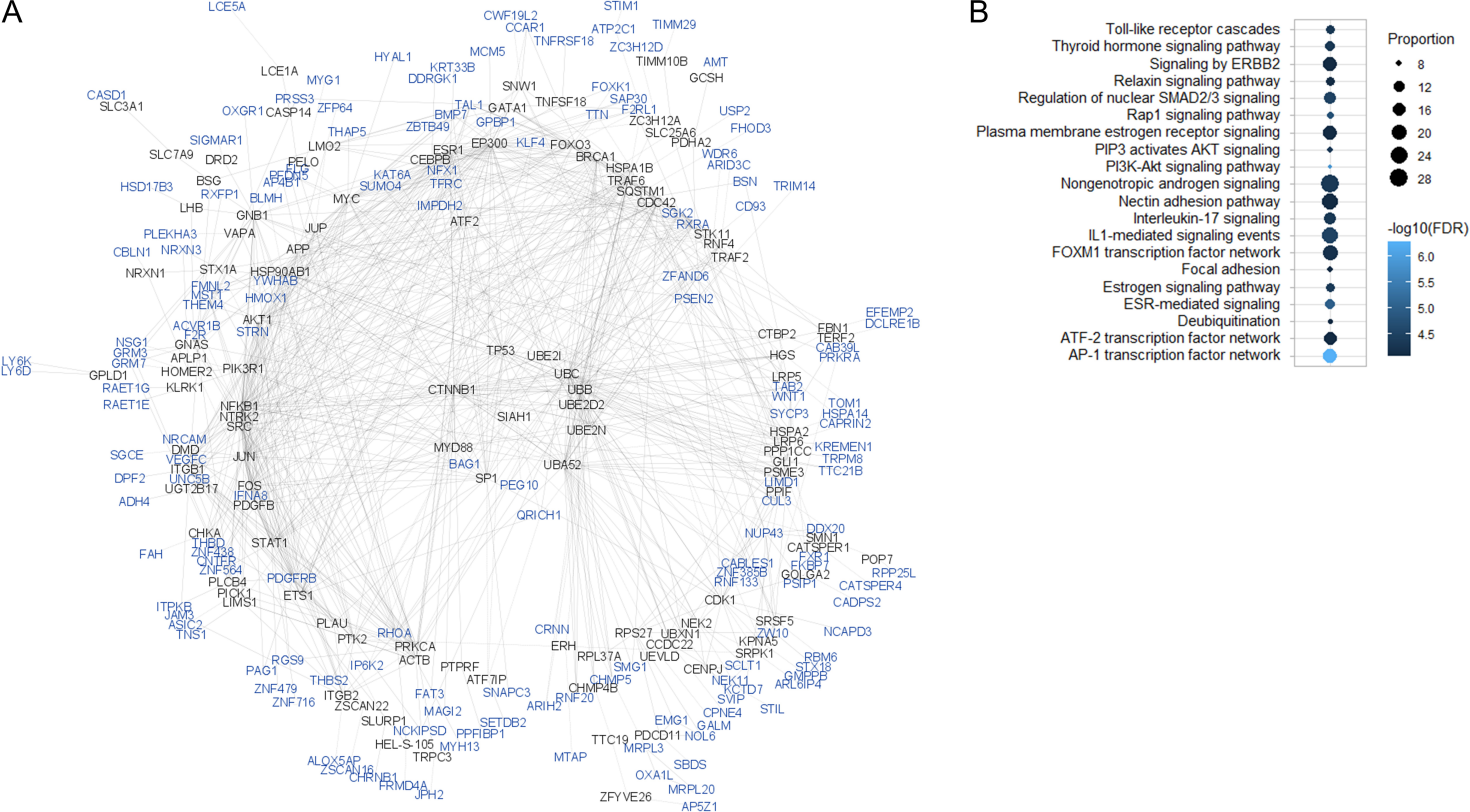
Integrated network analysis for atopic and autoimmune comorbidities in alopecia areata.**(A)** Protein-protein interaction network for comorbidities. The network was created using the ReactomeFIViz app for Cytoscape v.3.9.1 and 302 genes identified in the present analyses as input (i.e., genes mapped by location to supported loci + eQTL genes annotated to supported loci + MAGMA gene associations for all investigated phenotypes). From these, 116 protein products (blue) were integrated into the network using 96 non-input proteins as linkers (black). **(B)** Top 20 filtered pathways showing overrepresentation in the network. Circle size indicates the proportion (%) of network proteins belonging to the pathway term. Circle color denotes the statistical significance of the hypergeometric test, with lighter color indicating a higher level of significance (i.e. lower p-values). The top 100 filtered pathway terms (see: Methods) enriched in this network are presented in **Supplementary Table 4**. FDR: false discovery rate.

Next, the PPI network for comorbidities in general was created using the 302 genes identified in the analyses of all 10 investigated phenotypes as input, i.e., genes mapped by location to supported loci + eQTL genes annotated to supported loci + MAGMA gene associations. The resulting PPI network included 191 nodes, of which 95 were input proteins (**Figure 3A**). After filtering, the top enriched pathways in the comorbidities PPI network included i) networks for the transcription factors AP-1, ATF-2, and FOXM1; ii) immune pathways, such as signaling by interleukins (IL)-1 and -17, and toll-like receptors (TLRs); iii) thyroid and sex hormone signaling pathways; iv) phosphatidylinositol 3−kinase (PI3K)/protein kinase B (Akt) signaling pathway; and v) focal adhesion (**Figure 3B**). In addition, separate PPI networks were created for atopic and autoimmune comorbidities. These were generated from input lists containing 103 and 187 genes, respectively. The resulting PPI network for atopic comorbidities consisted of 120 nodes, of which 58 were input proteins (**Supplementary Figure 4**). The resulting PPI network for autoimmune comorbidities comprised 179 nodes, of which 93 were input proteins (**Supplementary Figure 5**). In the PPI network for atopic comorbidities, the most significant overrepresented pathways included relaxin signaling and inflammation pathways, such as TLR cascades and cytokine (e.g., IL-1/17, TNF) signaling. In the PPI network for autoimmune comorbidities, the top enriched pathways included signaling mediated by estrogen and vascular endothelial growth factor receptors, as well as PI3K-Akt signaling. The post-filtering top 100 overrepresented pathway terms in the generated PPI networks are shown in **Supplementary Table 4**. Within these lists, 26 pathway terms were enriched in all three PPI networks. Of these, the terms with the highest degree of overlap between PPI network nodes and pathway genes included nongenotropic androgen signaling, IL1-mediated signaling events, p53 pathway feedback loops, and the S1P3 pathway (**Supplementary Figure 6**).

## Discussion

This report presents an exploratory GWA study examining the development of atopic and/or autoimmune comorbidities in individuals with AA, with the goal of identifying genetic variants, genes, and biological pathways that predispose AA patients to multimorbidity with other immune-mediated diseases. The study included 2,332 AA patients of Central European ancestry, of whom 1,030 reported no atopic or autoimmune comorbidities and were classified as controls. Ten comorbid phenotypes were examined, encompassing (1) broader atopic/autoimmune comorbidity categories and (2) individual diseases, comprising atopic dermatitis, rhinitis, asthma, AITD in general, HT specifically, and vitiligo. Importantly, because the control group consisted of AA patients without comorbidities, the identified association signals should be interpreted as potential genetic modifiers influencing susceptibility to comorbid disease development within the AA population, rather than as general-population risk loci for these disorders.

In the absence of genome-wide significant association signals at the variant or gene level, we applied liberal thresholds established for exploratory analyses and identified genomic loci and genes that represent potential genetic contributors to comorbid CID development in the context of AA. To interpret these findings biologically, we integrated eQTL information from public resources and performed network analyses of functional interactions. These complementary approaches revealed that many suggestive loci harbor variants with known regulatory effects on gene expression in fibroblasts, skin, and immune cell populations, and that the identified genes cluster in functional networks related to sex and thyroid hormone signaling, proinflammatory cytokines, and pathways controlling cell growth and survival.

For the comorbidities (any) category, the top associated locus was located at chr13:50009874-50108857. This region encompasses seven genes. Of these, *PHF11*, *SETDB2*, and *RCBTB1* showed the largest numbers of mapped variants and known eQTLs. However, *PHF11* was the most frequent finding in our exploratory results overall. For *PHF11*, the present analyses revealed suggestive associations in AA for comorbid atopy and autoimmunity, in particular for atopic dermatitis and AITD, respectively. The *PHF11* gene encodes a plant homeodomain type zinc finger protein expressed in immune cells of relevance to both atopy and/or autoimmunity, i.e., T and B cells, NK cells, monocytes and dendritic cells. In T cells, *PHF11* was shown by previous research to play a role in Th1-type cytokine gene expression, particularly IFN-γ, by co-operating with NF-kB, and thus to contribute to T cell viability and activation (18, 19). In the context of atopy, a previous study of *PHF11* transgenic mice found that *PHF11* promoted class-switch recombination to IgE in activated B cells in a process that also involved NF-κB, exaggerated antigen-specific IgE production and allergic responses *in vivo* (20). *PHF11* has also been associated with total serum immunoglobulin E levels, and with atopic dermatitis in childhood (21, 22). An association with asthma has also been suggested although, to date, replication attempts have generated inconsistent results (23–26). Finally, besides its role in T and B cells, previous research has shown that *PHF11* is expressed in keratinocytes and may be a part of their innate immune response (27).

For atopic comorbidity development, the most frequently implicated gene in our gene-based GWA analyses, particularly for atopic dermatitis and asthma, was *ADH4*. This gene encodes the class II alcohol dehydrogenase 4 pi subunit. In the GWAS Catalog (www.ebi.ac.uk/gwas) (28), *ADH4* is associated with metabolic and blood chemistry traits, including serum lipids and sex hormone-binding globulin (SHBG) levels. SHBG is the main carrier of sex hormones in blood, and thereby controls their availability throughout the body. Alterations in SHBG levels have been observed in multiple medical conditions, including asthma (29, 30). Moreover, sex hormones exert regulatory effects on the immune response and the skin barrier, and have been implicated in the pathogenesis of atopic dermatitis (31). In addition, serum lipid levels have been reported to have possible causal effects in both asthma and atopic dermatitis (32). These associations raise the possibility that ADH4-related regulation of lipid metabolism or hormone transport might contribute to atopic comorbidity development in AA.

Within the suggestive loci for the atopic/autoimmune comorbidities (any) and autoimmune (only) phenotypes, the present analyses demonstrated a large number of eQTLs in fibroblasts, skin and blood, and – to a lesser extent – T cell subsets. Since genetic variants can have tissue-specific effects on the expression of multiple genes, these annotations help explain how a given genomic locus might link disorders that affect different tissues. For example, in the present AA cohort, variants mapping to *TAB2* exhibited suggestive associations with both autoimmune (only) comorbidities and asthma, emerging as the top locus in each phenotype. TGF-β activated kinase 1 (TAK1)-binding protein 2 (TAB2) regulates cell death signaling via the activation of TAK1. This in turn leads to activation of NF-κB and the transcription of prosurvival genes. During embryonic development, TAB2 is crucial for cardiac development, as well as for the prevention of apoptosis/necroptosis in the heart and other organs, such as the liver. In mice, TAB2 deficiency induces cardiac remodeling, heart failure, and pulmonary congestion (33). A considerable number of variants in the *TAB2* locus have regulatory effects on genes such as *TAB2 per se*, *LRP11*, *GINM1, RAET1E, RAET1G, NUP43*, and *PCMT1*, with tissue-specific patterns across skin, arterial tissue, and fibroblasts. For example, while the effects on *LRP11* and *PCMT1* are limited to certain regions of the skin and arterial tissues, respectively, *TAB2* is regulated in both skin and arterial tissues, and effects in fibroblasts are observed for *GINM1* only (see **Supplementary Figure 3**). In vitiligo and asthma, (dermal/lung) fibroblasts are key players in disease development (34, 35). The eQTL effects on *RAET1E* and *RAET1G*, also known as *ULBP4* and *ULBP5*, respectively, are of particular interest given the known association between *ULBP3/ULBP6* and AA development. ULPB gene cluster (*ULBP1-6*) encode for ligands of the NKG2D receptors that are expressed in NK cells, γδ T cells and also a subset of CD8+ T cells, namely, NKG2D+ CD8+ T cells, which play a crucial role in AA pathogenesis. *ULBP3/ULBP6* is the second strongest AA locus identified by GWA to date, and *ULPB3* was shown to be highly upregulated in the hair follicles of AA patients compared to unaffected controls (9). Variants at the *TAB2* locus showing suggestive association with the development of autoimmune comorbidities and asthma in our study are associated with an increased expression of *ULBP4* and *ULBP5* in the skin tissue.

In previous work, our group demonstrated that stop mutations in filaggrin (*FLG*) were associated with the presence of atopic dermatitis in patients with AA, and a more severe AA disease course in comorbid patients (36). Interestingly, genetic variants at the *LCE3E* locus that showed suggestive association with the development of comorbid atopic dermatitis in the present study regulate the expression of a cluster of genes in the epidermal differentiation complex that are crucial for skin barrier formation and integrity, including hornerin (*HRNR*), *FLG*, and cornulin (*CRNN*) (37).

In the present analyses, we also annotated the regulatory effects of the identified variants across various immune cell types. Variants showing suggestive associations with autoimmune comorbidity development in general displayed widespread eQTL effects across multiple immune cell populations. For the individual phenotypes, only a limited number of immune-cell annotations were observed that were restricted to eQTL effects in non-classical monocytes for AITD, Tfh cells for atopic dermatitis, and NK cells for vitiligo. We also observed that eQTL effects in classical monocytes were present for the atopic/autoimmune comorbidities (any) phenotype but absent for autoimmune (only) phenotypes, suggesting that the monocyte-related signal in the combined atopic/autoimmune category may primarily reflect contributions from atopic conditions. This interpretation would be consistent with reported evidence of changes in monocytes and macrophages in inflammatory skin diseases, including atopic dermatitis (38–40). Conversely, eQTL effects in B cells were detected specifically for autoimmune phenotypes and not for the comorbidities (any) phenotype, which aligns with the well-established role of B cells in autoimmunity (41).

Finally, network analyses of our integrated findings suggested interesting potential molecular mechanisms that may underlie the development of atopic and autoimmune comorbidities in patients with AA. These included thyroid and sex hormone signaling pathways, TLR cascades, proinflammatory cytokine signaling, and regulation of cell growth and survival. In the PPI network for the exploratory findings overall, the most significantly enriched pathway terms were the activator protein (AP)-1 transcription factor network, the PI3K-Akt signaling pathway, and estrogen receptor-mediated signaling. AP-1 is a key regulator of multiple cellular processes, including differentiation, proliferation, transformation, apoptosis, and migration. AP-1 activity is induced by cytokines, chemokines, growth factors, hormones, and environmental stressors. AP-1 is thus involved in the immune response, and has been implicated in the pathophysiology of diverse inflammatory conditions, including asthma (42). The PI3K-Akt pathway regulates processes such as cell proliferation, differentiation, migration, and apoptosis, as well as angiogenesis and metabolism. The PI3K-Akt pathway contributes to the development, function, and stability of regulatory T cells (43), as well as skin homeostasis, which implicates PI3K-Akt signaling in autoimmunity and skin disorders (44, 45). Estrogen exerts regulatory effects on cells of the innate and adaptive immune systems, including neutrophils, macrophages, and T and B cells. Estrogen receptors mediate the expression of estrogen-responsive genes, and are thus responsible for the control of estrogen-mediated Th1 and Th2 activities and the role of estrogen in autoimmune and inflammatory diseases (31, 46). Notably, the largest overlap between the present findings and the pathway gene lists was found for nongenotropic androgen signaling (28%). This pathway term refers to androgen effects that are considered nongenomic due to the fact that they occur in cells lacking a functional androgen receptor, occur in the presence of transcriptional/translational inhibitors, or are too rapid to involve transcriptional changes (47). Nongenomic immunosuppressive effects for testosterone are found in macrophages and T cells (48), and various studies have suggested the involvement of androgens in the development and course of atopic diseases (49). In addition to estrogen and androgens, signaling by glucocorticoids, relaxin, and thyroid and growth hormones were enriched in our PPI networks, and all of these factors have implications for immune regulation and immune-mediated disease (50–52). Therefore, our results suggest plausible immunoregulatory pathways as potential targets for the management of atopic and autoimmune comorbidities in patients with AA.

The present study had several limitations. First, the relatively small sample size limited the statistical power to detect robust association signals, and the small numbers of individuals with only one comorbidity hindered our ability to disentangle disease-specific effects. In addition, because ten primary GWA analyses were conducted across related phenotypes with partially overlapping case groups, we applied permissive significance thresholds appropriate for exploratory analyses and did not correct for the number of GWA analyses performed, warranting cautious interpretation of the results. Accordingly, although several of the identified loci and pathways show biological plausibility supported by existing literature, these findings should be considered hypothesis-generating and require validation by further research. Another limitation is that comorbidity information was obtained from patient self-report, which may introduce some degree of recall or reporting bias. At the same time, this approach allowed the systematic assessment of multiple comorbid conditions across the entire cohort and enabled the stratified analyses performed in this study. An additional limitation relates to the separate genotyping and imputation of the contributing datasets. However, we performed various rounds of QC to ensure high data quality and integrity, and demonstrated that no major bias concerns were present for the final merged dataset.

Despite these limitations, the comorbidity-stratified within-cohort GWA study design represents a notable methodological strength, enabling the detection of novel genomic association signals that cannot be resolved using genetic correlation-based approaches. More importantly, by utilizing the largest clinically and genetically characterized AA cohort assembled to date, this study provides, to our knowledge, the first genome-wide insights into comorbid CID development in AA. These findings provide a foundation for future mechanistic and large-scale genetic studies aimed at elucidating the molecular basis of multimorbidity in AA.

## Data Availability

The original contributions presented in the study are included in the article/**Supplementary Material**, further inquiries can be directed to the corresponding author.
