# Supplementary material for: Genome-wide association study of atopic and autoimmune comorbidities in alopecia areata
Source: Front Immunol. 2026 Apr 17;17:1810658. doi: 10.3389/fimmu.2026.1810658 (PMC13133067; doi:10.3389/fimmu.2026.1810658)
Supplement: Supplementary file 1 [file DataSheet1.pdf]

Genome-wide association study of atopic and autoimmune comorbidities in alopecia areata

Marisol Herrera-Rivero*, Yasmina Gossmann*, Swapnil Awasthi, Nicole Cesarato, Stephan Ripke, Bettina Blaumeiser[6], Ulrike Blume-Peytavi, Gerhard Lutz, Silke Redler, Markus M. Nöthen, Regina C. Betz, F. Buket Basmanav

SUPPLEMENTARY FIGURES

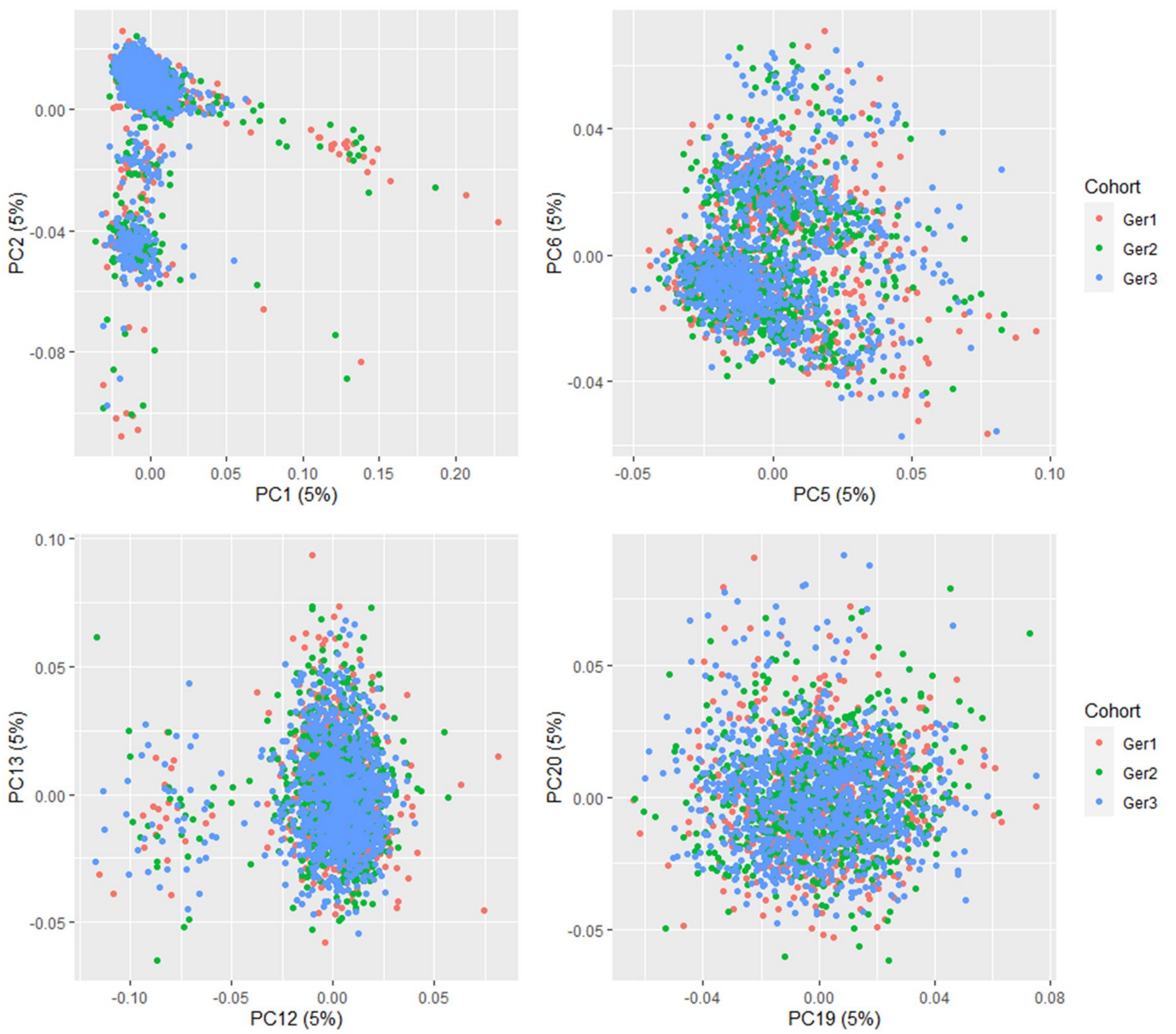

Figure S1. Representative plots of the principal components (PCs) analysis of genotypes. The observed proportion of variation within the study sample was small (5%). No effects of genotyping batch were observed. The first 20 PCs included as covariates in variant-based association analyses adequately addressed potential stratification issues.

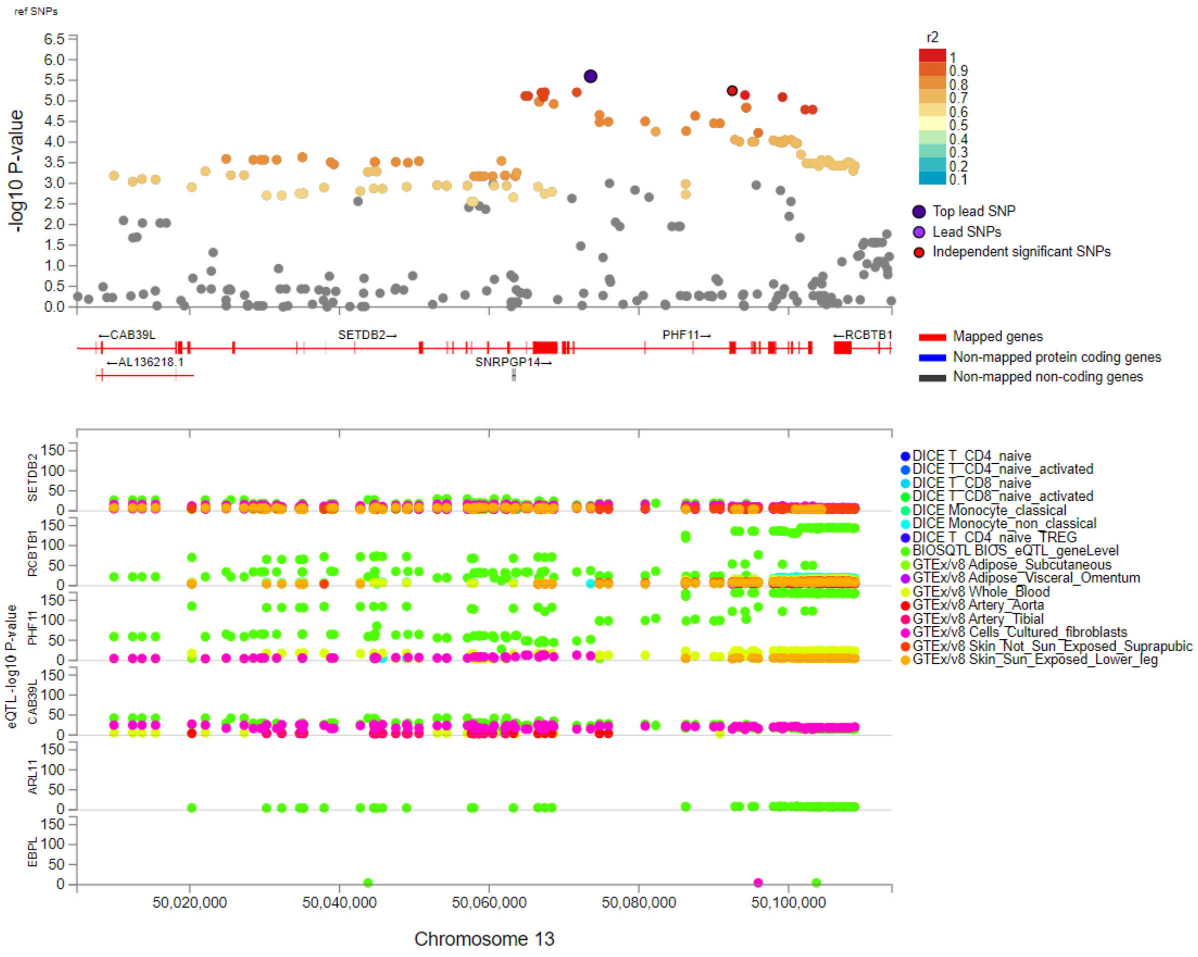

Figure S2. Top locus for atopic/autoimmune comorbidities (any) in alopecia areata. Genomic location: 13:50009874-50108857. This corresponds to the locus #5 identified for the atopic/autoimmune comorbidities (any) phenotype, consisting of 133 variants. Lead variant: rs3794381 ($p=2.5x10^{-6}$, odds ratio=1.4, mapped gene: *PHF11*). Independent significant variant: rs7329078 ($p=5.7x10^{-6}$, odds ratio=1.3, mapped gene: *PHF11*). The figure shows all genes mapped by location, as well as known expression quantitative trait loci (eQTLs) annotated to variants within the locus as queried from selected datasets using FUMA GWAS (see: Methods). At the gene-level, *PHF11* was the most widely suggested gene in our analyses.

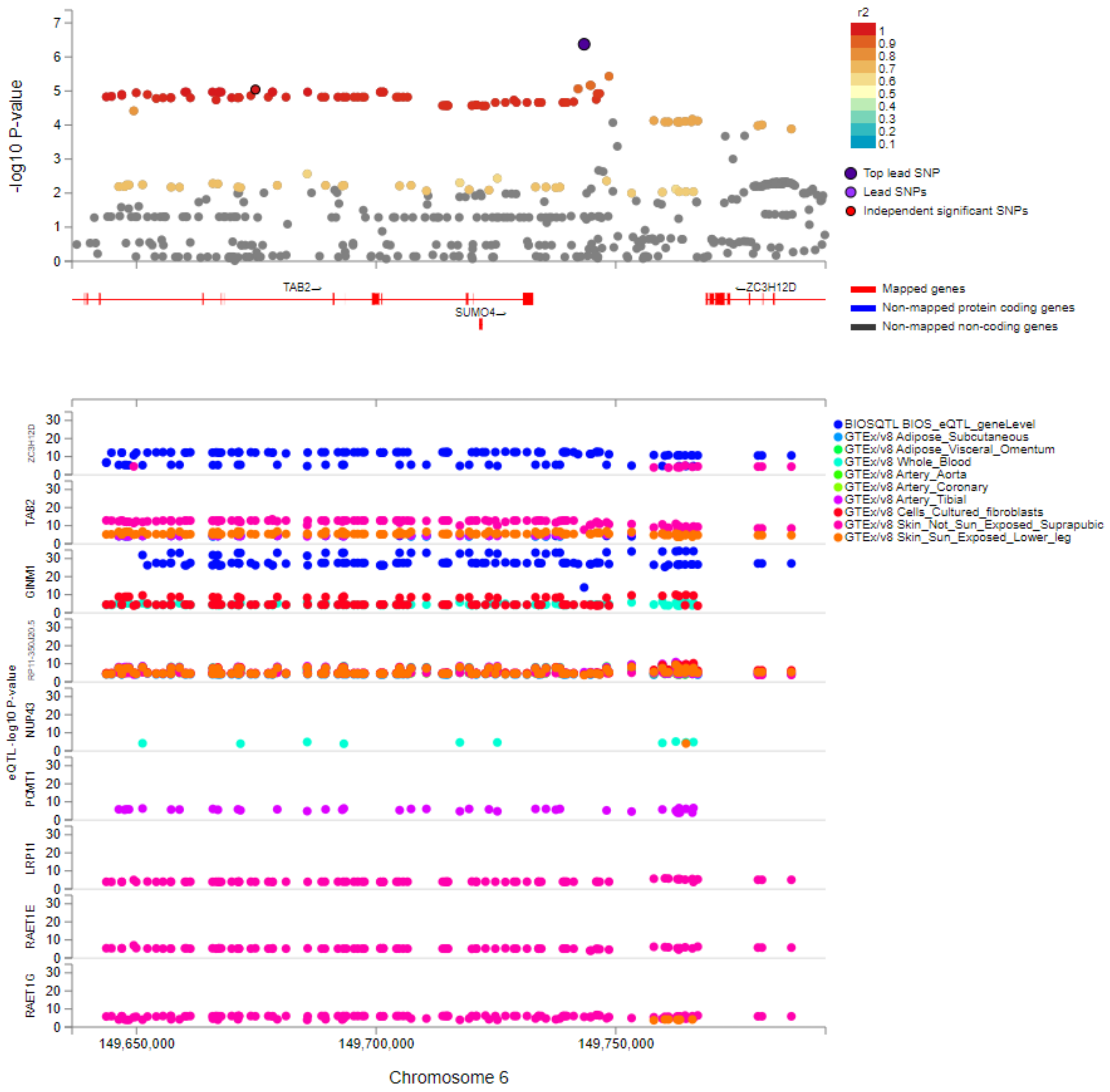

Figure S3. The shared locus for the autoimmune (only), atopic/autoimmune (any) and asthma phenotypes. Genomic location: 6:149643709-149786639. The regional association plot shows the locus #6 identified for the autoimmune (only) phenotype, which was the most significant locus for this phenotype, consisting of 125 variants (lead variant: rs6926771, $p=4.2x10^{-7}$, odds ratio=1.9, mapping: intergenic). The figure shows all genes mapped by location, as well as known expression quantitative trait loci (eQTLs) annotated to variants within the locus as queried from selected datasets using FUMA GWAS (see: Methods). From the three genes mapped within the region, *TAB2* contained the largest number of variants mapped by location (86) and with eQTL effects (122), mainly in skin tissues. In atopic/autoimmune comorbidities (any) and asthma phenotypes, the locus encompassed 98 variants due to the p-value threshold (i.e. less variants in the locus reached nominal significance for these phenotypes), from which 65 mapped to *TAB2* and 95 showed eQTL effects for this gene.

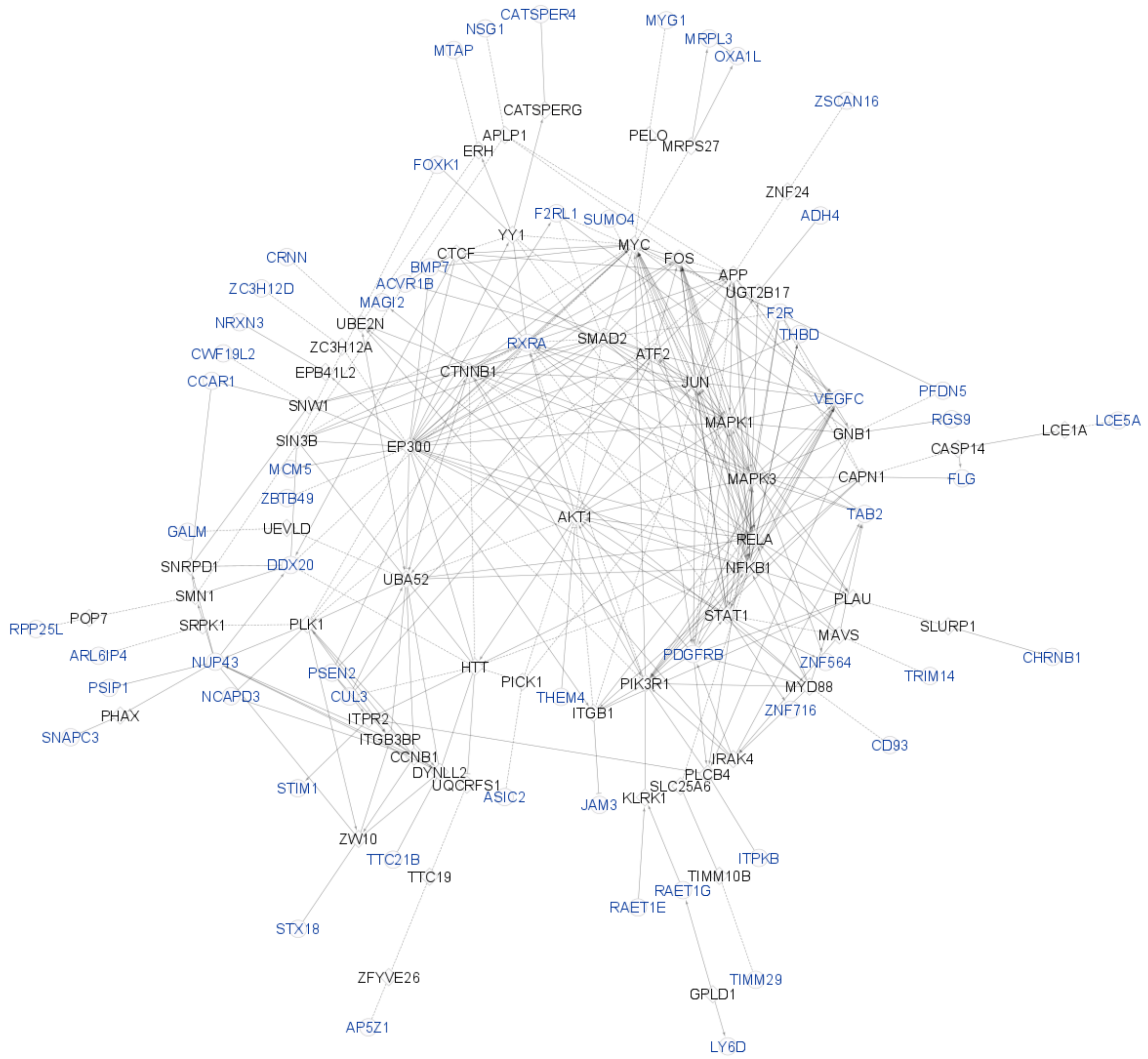

Figure S4. Protein-protein interaction network for atopic comorbidities. The network was created with the ReactomeFIViz app for Cytoscape v.3.9.1 using as input 103 genes resulting from our analyses (i.e. genes mapped by location to supported loci + eQTL genes annotated to supported loci + MAGMA gene associations) on atopic phenotypes (i.e. atopic only, atopic dermatitis, rhinitis and asthma). From these, 58 protein products (blue) were integrated into the network using 62 non-input proteins as linkers (black). The top 100 filtered pathway terms (see: Methods) enriched in this network are presented in the Supplementary Table 4, from which the most significant were the KEGG relaxin and toll-like receptor signaling pathways (false discovery rate=$1.4\text{x}10^{-8}$).

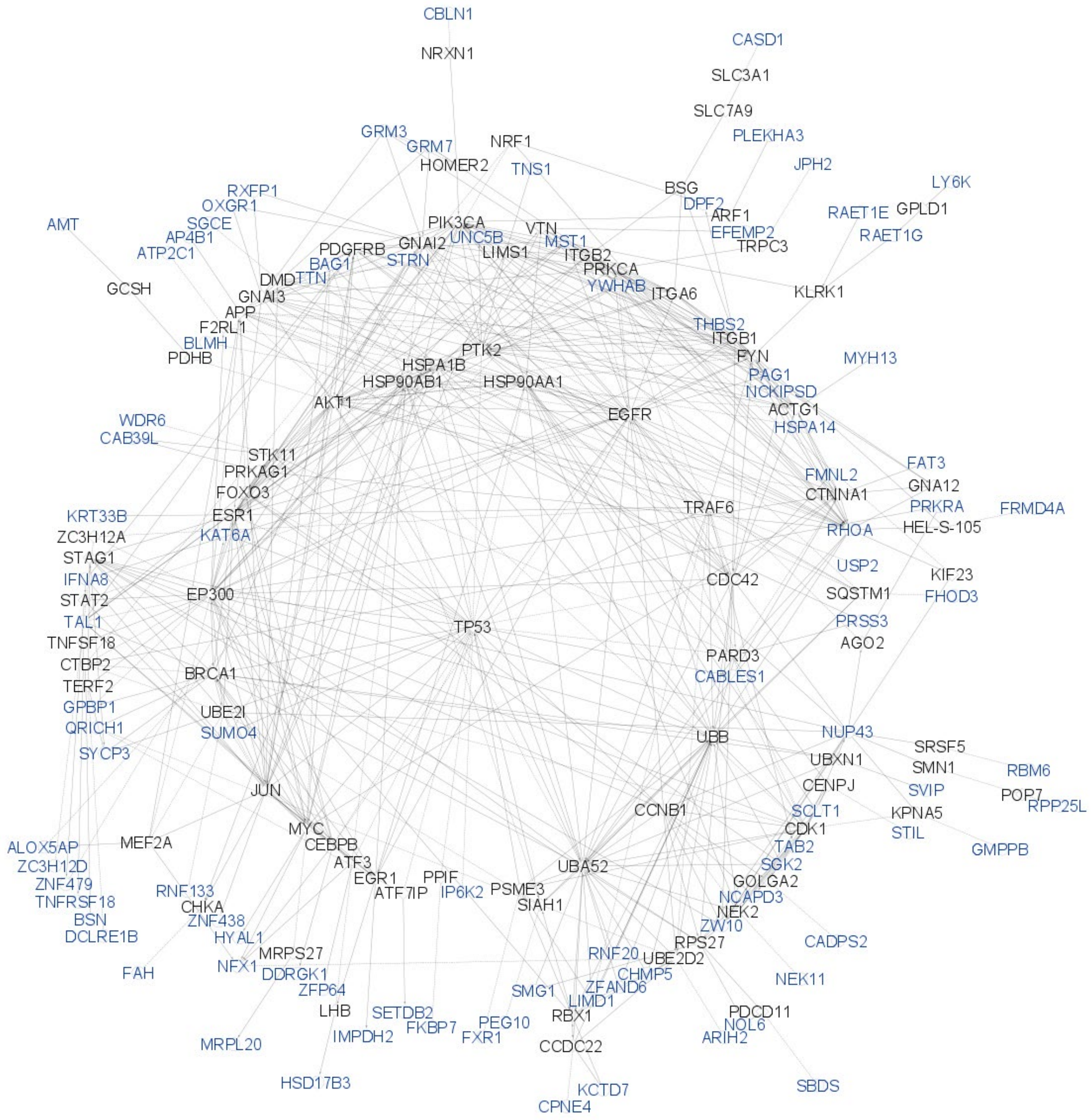

Figure S5. Protein-protein interaction network for autoimmune comorbidities. The network was created with the ReactomeFIViz app for Cytoscape v.3.9.1 using as input 187 genes resulting from our analyses (i.e. genes mapped by location to supported loci + eQTL genes annotated to supported loci + MAGMA gene associations) on autoimmune phenotypes (i.e. autoimmune only, vitiligo, autoimmune thyroid disease and Hashimoto's thyroiditis). From these, 93 protein products (blue) were integrated into the network using 86 non-input proteins as linkers (black). The top 100 filtered pathway terms (see: Methods) enriched in this network are presented in the Supplementary Table 4, from which the most significant were the Reactome's ESR-mediated signaling and NCI-Nature's Signaling events mediated by VEGFR1 and VEGFR2 pathways (false discovery rate=2.8x10^{-8}).

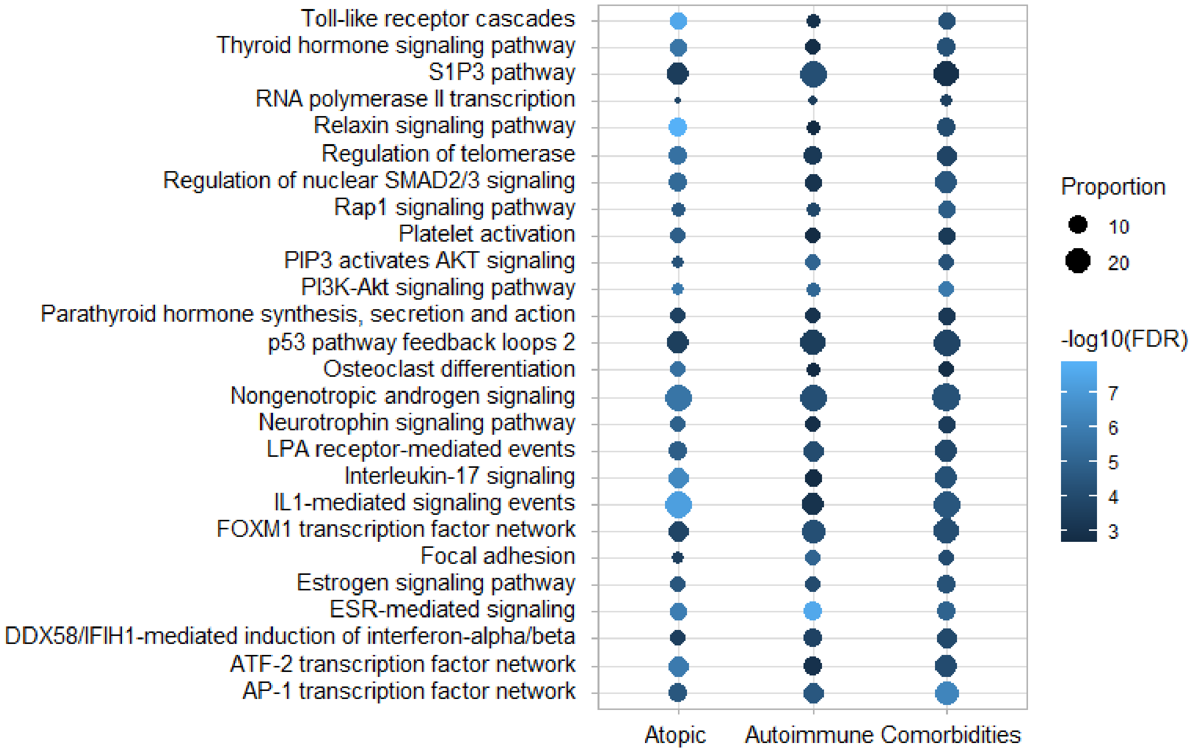

Figure S6. Pathways enriched in protein-protein interaction networks. Pathway terms that were enriched in all three networks are shown. The size of the circles represents the proportion (%) of network proteins belonging to the pathway term. The color of the circles denotes the statistical significance of the hypergeometric test, with higher significance (i.e. lower p-values) represented by lighter color. FDR: false discovery rate.
